# Self-Compatibility Alleviates Inbreeding Depression and Maintains Yield and Quality in Winter Turnip Rape (*Brassica rapa* L.)

**DOI:** 10.3390/plants15213475

**Published:** 2026-11-06

**Authors:** Jianzhong Sang, Xiaofang Ding, Jiquan Zhang, Bingyan Liu, Xiaoming Lu, Zhuanhong Liang, Tingting Fan, Lijun Liu, Yuanyuan Pu, Junyan Wu, Li Ma, Wangtian Wang, Gang Yang, Wancang Sun

**Affiliations:** 1 State Key Laboratory of Aridland Crop Science, College of Agronomy, Gansu Agricultural University, Lanzhou 730070, China; 15693312807@163.com (J.S.);; 2 Seed Industry Research Institute, Gansu Province University, Lanzhou 730070, China; 3 College of Life Science and Technology, Gansu Agricultural University, Lanzhou 730070, China

**Keywords:** winter turnip rape, self-compatibility, inbreeding depression, agronomic traits, physiological and biochemical characteristics

## Abstract

Winter turnip rape (*Brassica rapa* L.) is an important winter oilseed crop in the arid and cold regions of northern China due to its strong cold tolerance and wide adaptability. However, its prevalent self-incompatibility readily causes inbreeding depression, leading to declines in yield and quality and creating difficulties for cultivar breeding and pure-line propagation. To clarify how self-compatibility affects growth, yield, quality, and physiological traits, highly self-compatible (HSC), self-compatible (SC), and self-incompatible (SI) lines were compared under self-pollination and open pollination, and leaf antioxidant enzyme activities and endogenous hormone contents were measured. The results showed that the effects of selfing on seedling vegetative growth, agronomic traits, quality, and biomass were far smaller in HSC and SC lines than in SI lines, with inbreeding depression markedly alleviated. The mean inbreeding depression rates (IDRs) of major traits in HSC, SC, and SI lines were 4.32%, 8.14%, and 20.05%, respectively, the IDRs of effective silique number per plant were 3.03%, 5.22%, and 20.53%, and those of seed yield were 4.69%, 9.39%, and 27.90%. Line-level ANOVA and Tukey tests (three lines per group) confirmed that the IDRs of SI lines were significantly higher than those of HSC lines for all six core traits, with SC lines intermediate. Physiological and biochemical analyses indicated that HSC lines maintained higher SOD activity, and the contents of growth-promoting hormones, such as gibberellin (GA), cytokinin (CTK), and auxin (IAA), were generally higher than in SI lines, whereas CAT activity did not show a consistent advantage: in HSC lines CAT did not differ significantly between SP and OP (the two shared a significance group), and under open pollination SI-OP was grouped with HSC-OP and SC-OP. In contrast, after selfing, SI lines accumulated more abscisic acid (ABA), whereas the ethylene (ETH) differences were not consistently significant and the coordination of their antioxidant enzymes was altered rather than disrupted. Correlation analysis showed that the contents of growth-promoting hormones (GA, CTK, IAA, and BR) were significantly or highly significantly positively correlated with yield traits, such as seed yield per plant, effective silique number per plant, and thousand-seed weight, whereas ABA and ETH contents were highly significantly negatively correlated. A structural equation model, based on composite scores of hormones, enzymes, growth, and yield and quality, indicated that the hormone composite was strongly associated with the growth composite (standardized path coefficients of hormone → growth and growth → yield and quality were 0.816 and 0.535, respectively), whereas the enzyme composite was directly associated with yield and quality (0.291). These paths reflect statistical associations among the composite scores rather than confirming the specific underlying mechanisms, and vegetative growth was the main mediator linking the hormone composite to yield and quality. To our knowledge, self-compatibility in winter turnip rape can maintain relatively stable plant growth and development as well as yield and quality by alleviating inbreeding depression and maintaining physiological metabolism and hormone homeostasis. This study provides a theoretical basis for the screening and utilization of self-compatible germplasm and for high-yield breeding of winter turnip rape; because stress tolerance was not directly measured, any extrapolation to stress-resistant breeding requires dedicated stress-tolerance experiments in future work.

## 1. Introduction

Rapeseed (*Brassica* spp.) is one of the world’s most important sources of vegetable oil and protein-rich feed meal. China is both a major producer and a major consumer of rapeseed [1,2]. Within this broad rapeseed sector, winter turnip rape is a regionally important crop adapted to the cold and arid north. Winter turnip rape has strong cold tolerance and wide adaptability. Its successful cultivation in the arid and cold regions of northern China has expanded the winter rapeseed planting area, serving both as winter vegetation cover and as edible oil production [3]. However, the vast majority of turnip rape germplasm exhibits self-incompatibility (SI): under bagged self-pollination, pollen germination and pollen tube elongation are impeded, resulting in low seed-setting rates. Nevertheless, in breeding practice repeated bagged self-pollination is deliberately imposed even on SI plants, because genetically homozygous parental lines are required for hybrid cultivar development. Since only a small fraction of self-pollinations succeed, the few selfed progenies obtained must be re-selfed over successive generations. Continuous selfing induces inbreeding depression, leading to declines in yield, quality, and stress resistance, and increases the difficulty of breeding genetically homozygous parental lines [4,5].

Currently, plant self-incompatibility systems are mainly divided into two categories: gametophytic self-incompatibility (GSI) and sporophytic self-incompatibility (SSI), which differ in genetic control and molecular mechanisms [6,7]. Brassicaceae plants exhibit SSI controlled by multiple genes at the S locus: after self-pollination, the stigma receptor kinase (S-locus receptor kinase, SRK) interacts with and recognizes the pollen coat ligand SCR/SP11, activating a downstream signaling cascade that rejects self-pollen and inhibits pollen hydration and germination [8,9,10]. Self-compatibility (SC) usually arises from loss-of-function mutations, structural variations, or transcriptional silencing of S-locus genes or their downstream factors. Natural SC mutants have been identified in *Arabidopsis thaliana*, *Brassica napus*, and turnip rape [9,10]. Beyond Brassicaceae, the molecular basis of SC has also been clarified in other crops: in diploid potato, a promoter insertion that causes neofunctionalization of the S-locus inhibitor (*Sli*) gene enables self-pollen acceptance and has become the key to diploid hybrid potato breeding [11].

Screening of turnip rape germplasm from different ecological regions has continuously revealed SC variation, such as Indian yellow sarson and some local cultivars and breeding lines in China [12,13]. Analogous S-genotype identification and compatible-resource exploration have also been conducted in fruit crops such as citrus [14]. In the arid and cold regions of northwestern China, systematic screening of strong-winter turnip rape has yielded dozens of SC materials, which have been classified into three types—highly self-compatible (HSC), self-compatible (SC), and self-incompatible (SI)—based on the relative self-compatibility index [15,16,17]. Field identification, fluorescence observation of pollen–stigma interactions, and S-locus molecular markers are often combined for the identification of SC germplasm [16,17]. With the rapid development of genomics, PCR-based molecular markers have been increasingly used to identify SC germplasm. Single-nucleotide polymorphism (SNP) markers, including kompetitive allele-specific PCR (KASP) and high-resolution melting (HRM) assays, allow high-throughput and precise genotyping of SC-related mutations, such as those in MLPK, whereas simple sequence repeat (SSR) and sequence-characterized amplified region (SCAR) markers provide stable, low-cost tools for marker-assisted selection [18,19,20].

Inbreeding depression refers to the decline in viability, fertility, yield, and stress resistance after continuous selfing, and is widespread in predominantly outcrossing species, including selfed progenies of hybrid rice [21]; genetically, it can be explained by the exposure of recessive deleterious alleles (dominance hypothesis) and the loss of heterozygote advantage (overdominance hypothesis) [22]. In Brassicaceae oil crops, selfing can cause depression in stress resistance and yield-related traits, and the magnitude of depression increases with the number of selfing generations [23,24,25]; conversely, inter-line hybridization can produce significant heterosis and has been used for seed yield improvement [26]. Meanwhile, quantitative trait loci (QTL) controlling SC, silique traits, and seeds per silique have been mapped, suggesting potential for the synergistic improvement of SC and yield component traits [27].

At the physiological level, selfing is often accompanied by reduced activities of antioxidant enzymes, such as superoxide dismutase (SOD), catalase (CAT), and peroxidase (POD), weakened reactive oxygen species (ROS) scavenging capacity, and membrane damage, whereas SC lines can maintain relatively stable antioxidant capacity [23,28]. Endogenous hormones also participate in pollen–stigma interactions and reproductive development: in selfed or SI plants, the contents of gibberellin (GA), cytokinin (CTK), and auxin (IAA) are often low, whereas those of abscisic acid (ABA) and ethylene (ETH) are high. This hormone imbalance is associated with inhibited pollen tubes, accelerated senescence, and suppressed growth [29]. Previous studies have shown that SC lines of winter turnip rape have higher selfed seed-setting rates, stronger cold tolerance, and more stable yields than SI lines [26]. The response of sarson-type turnip rape yield to cultivation measures also suggests that SC germplasm is suitable for stable-yield cultivation [30]. However, the above antioxidant enzymes and endogenous hormones do not act in isolation; they form complex coupling and cascade networks with agronomic, yield, and quality traits, and simple analysis of variance and pairwise comparisons cannot distinguish the direct and indirect effects of each factor. Correlation analysis and structural equation modeling (SEM) can decompose direct and indirect effects and identify mediating variables in multivariate networks and have been widely used to dissect source–sink relationships, yield formation, and physiological regulatory pathways in crops [31,32]. Nevertheless, research on the “hormone–antioxidant–growth–yield and quality” regulatory network in winter turnip rape under differences in self-compatibility remains lacking.

However, for strong-winter turnip rape with different self-compatibility, quantitative comparisons across the entire growth period—from seedling vegetative growth, agronomic traits, seed quality, and biomass to antioxidant and hormone responses—are still lacking. Accordingly, the present study used 9 lines representing the HSC, SC, and SI types, each evaluated under self-pollination and open pollination (18 line–treatment combinations), and compared, under bagged self-pollination and open pollination: (1) seedling, agronomic, quality, and biomass traits, (2) the inbreeding depression rate (IDR) of each trait, (3) SOD, POD, and CAT activities and the contents of six endogenous hormones, and (4) the coupling relationships among agronomic, quality, antioxidant enzyme, and endogenous hormone traits and their direct and indirect regulatory pathways, using Pearson correlation analysis and structural equation modeling. The aim was to determine whether and to what extent self-compatibility alleviates inbreeding depression in growth, yield, and quality, and to reveal its physiological basis and yield-quality regulatory network, thereby providing a basis for the utilization of highly self-compatible germplasm in winter turnip rape breeding.

## 2. Results and Analysis

### 2.1. Effects of Self-Compatibility on Growth, Development, and Quality of Winter Turnip Rape

#### 2.1.1. Effects of Selfing on Seedling Vegetative Growth in Lines with Different Self-Compatibility

**Figure 1:**
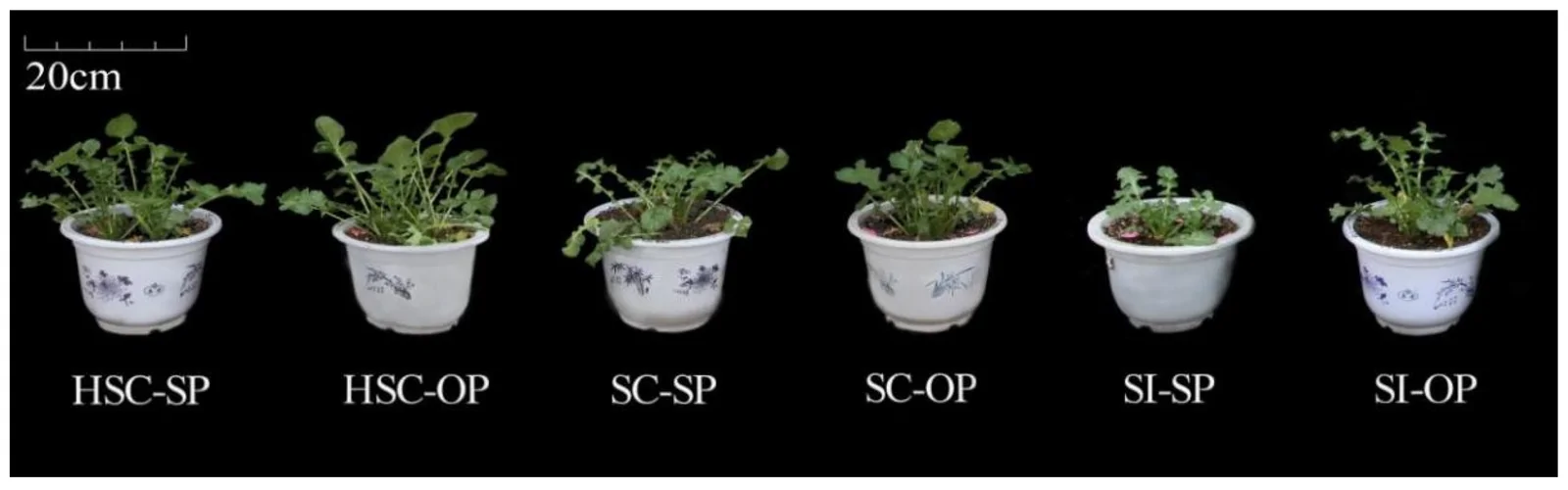
Seedling morphology of HSC, SC, and SI lines under self-pollination (SP) and open pollination (OP). Scale bar = 20 cm.

Leaf length, leaf width, and leaf number were measured for HSC, SC, and SI lines under bagged self-pollination (SP) and open pollination (OP; Table 1). The results showed significant differences in seedling leaf traits among lines with different self-compatibility (*p* < 0.05), with an overall gradient of HSC > SC > SI (Figure 1). HSC-OP lines had the highest leaf length (4.89 cm), leaf width (4.38 cm), and leaf number (5.72 leaves). SI-SP lines had the lowest values for all traits, with leaf length of only 2.76 cm, leaf width of 2.54 cm, and leaf number of 4.56, significantly different from HSC lines.

Coefficient of variation (CV) analysis showed that the CV of each trait ranged from 11.20% to 40.11%, with leaf number having the largest CV (40.11% in SI-OP) and leaf width being relatively stable (only 11.20% in SC-OP). Within the same line, the CV of traits in open-pollinated plants was generally higher than in selfed plants, indicating greater genetic dispersion in the outcrossing population.

Inbreeding depression rate (IDR) analysis showed that seedling traits of SI lines suffered the most severe inbreeding depression, with IDR for leaf length, leaf width, and leaf number reaching 25.81%, 22.32%, and 13.31%, respectively, all significantly higher than those of HSC lines (3.68%, 7.99%, 3.50%) and SC lines (5.59%, 9.29%, 6.01%). The IDR of all traits in HSC and SC lines was below 10%, indicating strong selfing tolerance.

#### 2.1.2. Effects of Selfing on Major Agronomic Traits in Lines with Different Self-Compatibility

Significant differences in major agronomic traits at maturity were observed among lines with different self-compatibility (*p* < 0.05; Table 2). An obvious gradient was evident overall: HSC lines performed best, SC lines were intermediate, and SI lines were poorest. Meanwhile, within the same line, the mean trait values under open pollination (OP) were generally higher than under self-pollination (SP). Specifically, plant-type-related traits, such as plant height, branching point height, and number of primary branches, as well as seed-setting-related traits, such as effective silique number per plant and silique length, were all significantly better in HSC lines than in SC and SI lines. In contrast, SI lines had the lowest mean values for all traits, especially after selfing treatment, which affected all compatible lines to varying degrees.

Analysis of the coefficient of variation (CV) and inbreeding depression rate (IDR) for each agronomic trait showed that trait stability and selfing tolerance were closely related to self-compatibility. In terms of CV, the agronomic trait CVs of SI lines were generally higher than those of HSC and SC lines. The CV of seeds per silique under SI-SP was as high as 82.58%, indicating the poorest trait stability, whereas the CVs of HSC lines were overall low, with the CV of thousand-seed weight under HSC-OP being only 0.73%, indicating the best trait stability. The IDR showed that the lower the self-compatibility, the more severe the depression: the IDR of HSC lines ranged from 3.03% to 14.07% (mildest inbreeding depression), that of SC lines ranged from 4.33% to 23.12% (moderate depression), and that of SI lines ranged from 7.19% to 38.57%, with the highest depression rates for branching point height and number of primary branches.

#### 2.1.3. Effects of Selfing on Seed Quality Traits in Lines with Different Self-Compatibility

Significant differences in seed quality indicators were observed among lines with different self-compatibility (*p* < 0.05; Table 3). Core quality indicators, such as protein, oil content, oleic acid, and linolenic acid, were generally better in HSC lines than in SC and SI lines; however, this pattern was driven mainly by the self-pollinated SI-SP group, which had the lowest values for most indicators, whereas under open pollination the SI-OP values were grouped with those of HSC-OP and SC-OP (e.g., oil content 44.45% and glucosinolates 107.82 µmol/g) and were not the lowest. HSC-OP lines had an oil content of 44.48% and protein content of 27.50%. SI-SP lines had an oil content of only 38.15% and protein content of 22.25%, significantly different from HSC lines.

Analysis of the coefficient of variation (CV) and inbreeding depression rate (IDR) for each quality indicator showed that the stability and selfing tolerance of quality indicators were closely related to self-compatibility. In terms of CV, the CVs varied markedly with the quality indicator and treatment rather than being consistently higher in any one line. For oil content, the CV was highest under SI-OP (4.57%) and lowest under HSC-SP (1.00%); in contrast, for oleic acid the CV was lowest under SI-SP (2.23%) and highest under HSC-OP (10.82%), and for protein the SI-SP CV (2.38%) was lower than those of HSC-SP (4.11%) and SC-SP (3.77%). Thus, no single line showed uniformly high or low CVs across all quality indicators. The IDR followed the pattern that the lower the self-compatibility, the more severe the depression: the IDR of HSC lines ranged from 1.31% to 11.44% (mildest), that of SC lines ranged from 4.24% to 14.69% (moderate), and that of SI lines ranged from 5.44% to 16.53%, with the IDR of linolenic acid and glucosinolates both exceeding 16%, indicating the most severe inbreeding depression. Overall, the lower the self-compatibility, the stronger the inhibitory effect of selfing on quality traits.

#### 2.1.4. Effects of Selfing on Biomass in Lines with Different Self-Compatibility

Significant differences in biomass-related indicators were observed among rapeseed lines with different self-compatibility (*p* < 0.05; Table 4). Selfing treatment (SP) reduced the biomass of each plant part in all lines to varying degrees: within the same line, the mean biomass of each plant part under OP was higher than under SP, and the lower the self-compatibility, the more severe the inbreeding depression.

In terms of overall biomass performance, a gradient of HSC > SC > SI was observed. HSC lines had the highest mean values for grain, stem, pod shell, and root weight; under HSC-OP, grain (17.26 g), stem (21.08 g), pod shell (10.86 g), and root weight (3.56 g) were all the highest among all treatments. SI lines had the lowest biomass indicators; under SI-SP, grain (7.60 g), stem (11.94 g), pod shell (5.61 g), and root weight (1.84 g) were all the lowest, showing highly significant differences from HSC lines.

In terms of coefficient of variation (CV), there were obvious differences in the stability of biomass traits among different lines and treatments. The CVs depended strongly on the treatment and plant part: the highest CVs occurred for root weight under SC-SP (37.13%) and SC-OP (33.86%), indicating poor stability of root weight in SC lines; however, HSC lines were not uniformly stable, because the root-weight CV under HSC-OP (22.47%) was comparable to that of stem weight under SI-OP (23.47%). The SI lines did not show intermediate, consistent CVs—their CVs differed greatly among plant parts (e.g., 2.36% for grain and 11.17% for stem under SI-SP, and 7.01% for root and 23.47% for stem under SI-OP).

Inbreeding depression rate (IDR) analysis showed that self-compatibility was negatively correlated with the degree of inbreeding depression: the IDR of HSC lines ranged from 1.97% to 10.62% (mildest), with the IDR of pod shell being only 1.97% (least affected by selfing), that of SC lines ranged from 9.39% to 19.12% (moderate), with the highest IDR for stem (19.12%), and that of SI lines ranged from 27.90% to 41.77% (most severe), with the IDR of root weight reaching 41.77% and that of stem being 35.11%, indicating that self-incompatible lines have extremely poor tolerance to selfing and that selfing treatment significantly inhibits their biomass formation.

#### 2.1.5. Comprehensive Comparison of Inbreeding Depression Rates of Major Traits in Lines with Different Self-Compatibility

The IDRs of six core traits—plant height, effective silique number per plant, thousand-seed weight, oil content, seed yield, and biomass—were summarized (Table 5). One-way ANOVA on line-level IDR (3 lines per group; 18 line–treatment combinations) showed significant overall differences among the HSC, SC, and SI groups for all 6 traits (*p* < 0.05), and the IDRs of all traits followed the order SI > SC > HSC. For plant height, HSC and SC lines had similarly low IDRs (3.37% and 4.33%, not significantly different), whereas SI lines had a significantly higher IDR (7.19%). For effective silique number per plant, HSC and SC lines did not differ significantly (3.03% and 5.22%), while SI lines showed a significantly higher IDR (20.53%). For thousand-seed weight, HSC and SC lines were comparable (5.05% and 7.22%, not significantly different), and both were significantly lower than SI lines (15.64%). For oil content, the IDR was lowest in HSC (3.51%) and significantly lower than in SI (14.17%), with SC intermediate (8.38%, not significantly different from either). For grain weight, HSC (4.69%) was significantly lower than SI (27.90%), with SC (9.39%) intermediate and not significantly different from either. For biomass, the IDR in HSC (6.29%) was significantly lower than in SI (34.85%), while SC (14.30%) was intermediate and did not differ significantly from either group. Thus, the robust statistical result was that SI lines suffered significantly greater inbreeding depression than HSC lines for all six core traits, with SC lines generally intermediate.

Comprehensive analysis showed that the effects of selfing on seedling vegetative growth, agronomic traits, quality, and biomass were far smaller in self-compatible lines than in self-incompatible lines, with inbreeding depression markedly alleviated.

### 2.2. Effects of Self-Compatibility on Physiological and Biochemical Characteristics of Winter Turnip Rape

#### 2.2.1. Differences in Antioxidant Enzyme Activities Among Lines with Different Self-Compatibility

**Figure 2:**
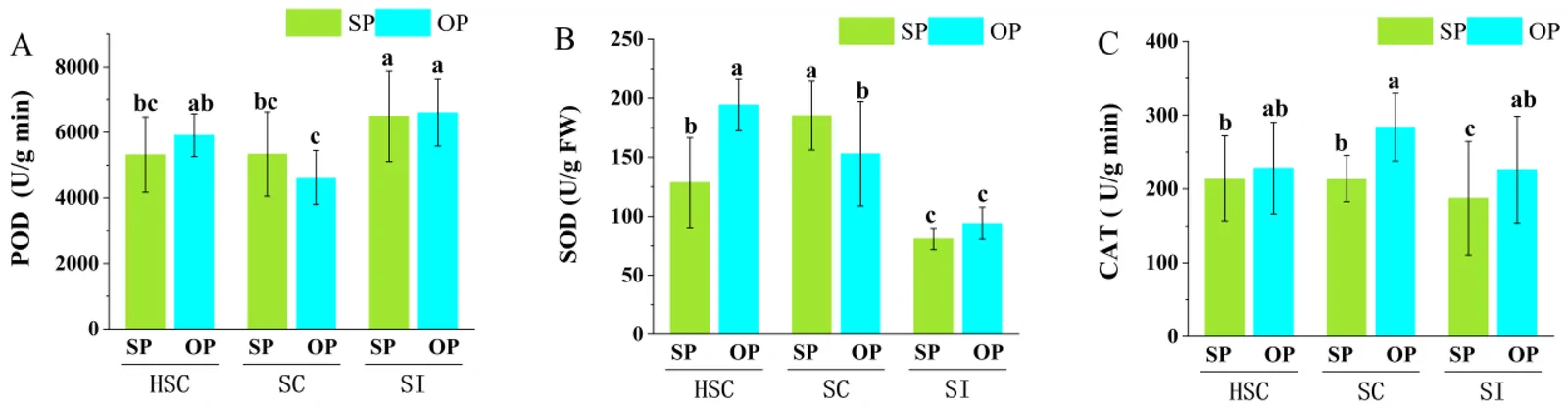
Activities of POD (**A**), SOD (**B**), and CAT (**C**) in leaves of HSC, SC, and SI lines under self-pollination (SP) and open pollination (OP). Different letters indicate significant differences (*p* < 0.05, Duncan’s test).

The activities of three antioxidant enzymes—POD, SOD, and CAT—were determined in HSC, SC, and SI lines under SP and OP treatments (Figure 2). The results showed significant differences in antioxidant enzyme activities among materials with different compatibility (*p* < 0.05).

POD activity was highest in SI lines, reaching 6495.22 and 6598.67 U·g^−1^·min^−1^ under SP and OP, respectively. SI-SP was significantly higher than HSC-SP (5318.77 U·g^−1^·min^−1^), but under open pollination SI-OP (6598.67) and HSC-OP (5912.22 U·g^−1^·min^−1^) belonged to the same statistical group and did not differ significantly. SOD activity was highest under HSC-OP (194.36 U·g^−1^·FW), significantly higher than under HSC-SP (128.61 U·g^−1^·FW). CAT activity was highest under SC-OP (283.91 U·g^−1^·min^−1^), but under open pollination HSC-OP, SC-OP, and SI-OP fell within the same statistical group; only the self-pollinated SI-SP group was significantly lowest, whereas SI-OP recovered to the same statistical group as HSC and SC.

Different pollination methods had clearly different regulatory effects on the antioxidant enzyme system of each line. In HSC lines, SOD activity was significantly higher under OP than under SP, whereas CAT activity did not differ significantly between OP and SP (the two shared the same significance group), indicating that under open pollination, HSC lines enhance ROS scavenging mainly through SOD rather than through CAT. In SI lines, POD activity was high under both treatments (and was grouped with HSC-OP under OP), SOD was the lowest under both treatments, and CAT was lowest under SI-SP but recovered under SI-OP to the same statistical group as HSC and SC, so CAT was not consistently low. These results indicate altered rather than disrupted coordination of the antioxidant enzymes. The idea that the single-POD pattern reflects persistent oxidative stress is a hypothesis that would require direct ROS or oxidative-damage measurements.

#### 2.2.2. Characteristics of Endogenous Hormone Content Changes in Lines with Different Self-Compatibility

**Figure 3:**
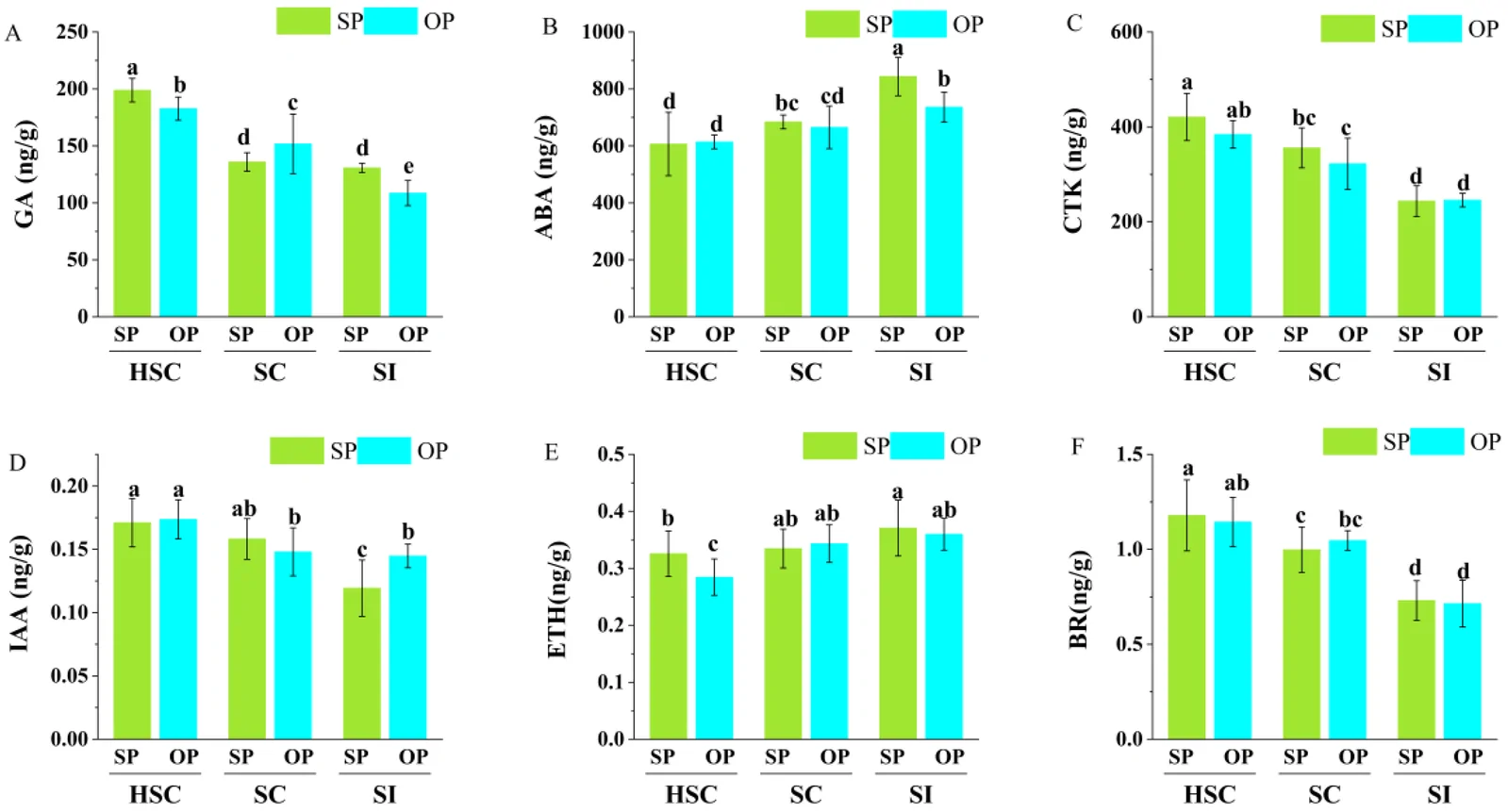
Endogenous hormone contents in leaves of HSC, SC, and SI lines under self-pollination (SP) and open pollination (OP): GA (**A**), ABA (**B**), CTK (**C**), IAA (**D**), ETH (**E**), and BR (**F**). Different letters indicate significant differences (*p* < 0.05, Duncan’s test).

The determination of the contents of six endogenous hormones (Figure 3) showed significant differences in hormone levels among materials with different compatibility (*p* < 0.05).

The growth-promoting hormones generally tended to be higher in HSC and lower in SI, but not every comparison was statistically significant: for CTK, HSC-SP, HSC-OP, and SC-SP shared a statistical group, for IAA, HSC-SP, HSC-OP, and SC-SP were grouped together, and for BR, HSC-SP, HSC-OP, and SC-OP were in the same group. The GA contents in HSC lines were 198.87 and 182.70 ng·g^−1^ under SP and OP, respectively, significantly higher than in SI lines (130.00 and 110.00 ng·g^−1^). The CTK contents in HSC lines were 420.89 and 384.27 ng·g^−1^, whereas those in SI lines were only 243.76 and 245.84 ng·g^−1^. The IAA content in HSC lines (0.17 µmol·g^−1^) was significantly higher than in SI lines (0.11–0.14 µmol·g^−1^). The BR content in HSC lines (1.17 and 1.14 pmol·g^−1^) was also significantly higher than in SI lines (0.73 and 0.71 pmol·g^−1^).

Growth-inhibiting hormones (ABA and ETH) showed the opposite trend. The ABA contents in SI lines reached 843.32 and 736.22 ng·g^−1^ under SP and OP, respectively, significantly higher than in HSC lines (606.63 and 613.92 ng·g^−1^). For ETH, SI-SP (0.37 pmol·g^−1^) was significantly higher than HSC-SP (0.32) and HSC-OP (0.28), but SI-OP (0.35) was in the same statistical group as HSC-SP (0.32), so the ETH difference between SI and HSC was not consistently significant.

Regarding the effects of pollination method, in HSC lines under SP, GA remained at the highest level (group a), whereas CTK and BR were in the same statistical group under SP and OP (i.e., they were not specifically elevated under SP), and ABA did not accumulate significantly, indicating that highly compatible materials can maintain the dominance of growth-promoting hormones and hormone balance under selfing conditions. In SI lines, GA and ABA instead increased under SP, whereas CTK and BR were both in the lowest group and did not differ between SP and OP, and IAA was also in a single group (b) under both treatments (but was not the lowest, since SC-OP was lower), indicating that the hormone regulatory system of self-incompatible materials responds sluggishly to changes in pollination method and cannot alleviate selfing stress through effective regulation of hormone levels.

### 2.3. Correlation Analysis of Agronomic, Quality, Physiological, and Hormone Traits

**Figure 4:**
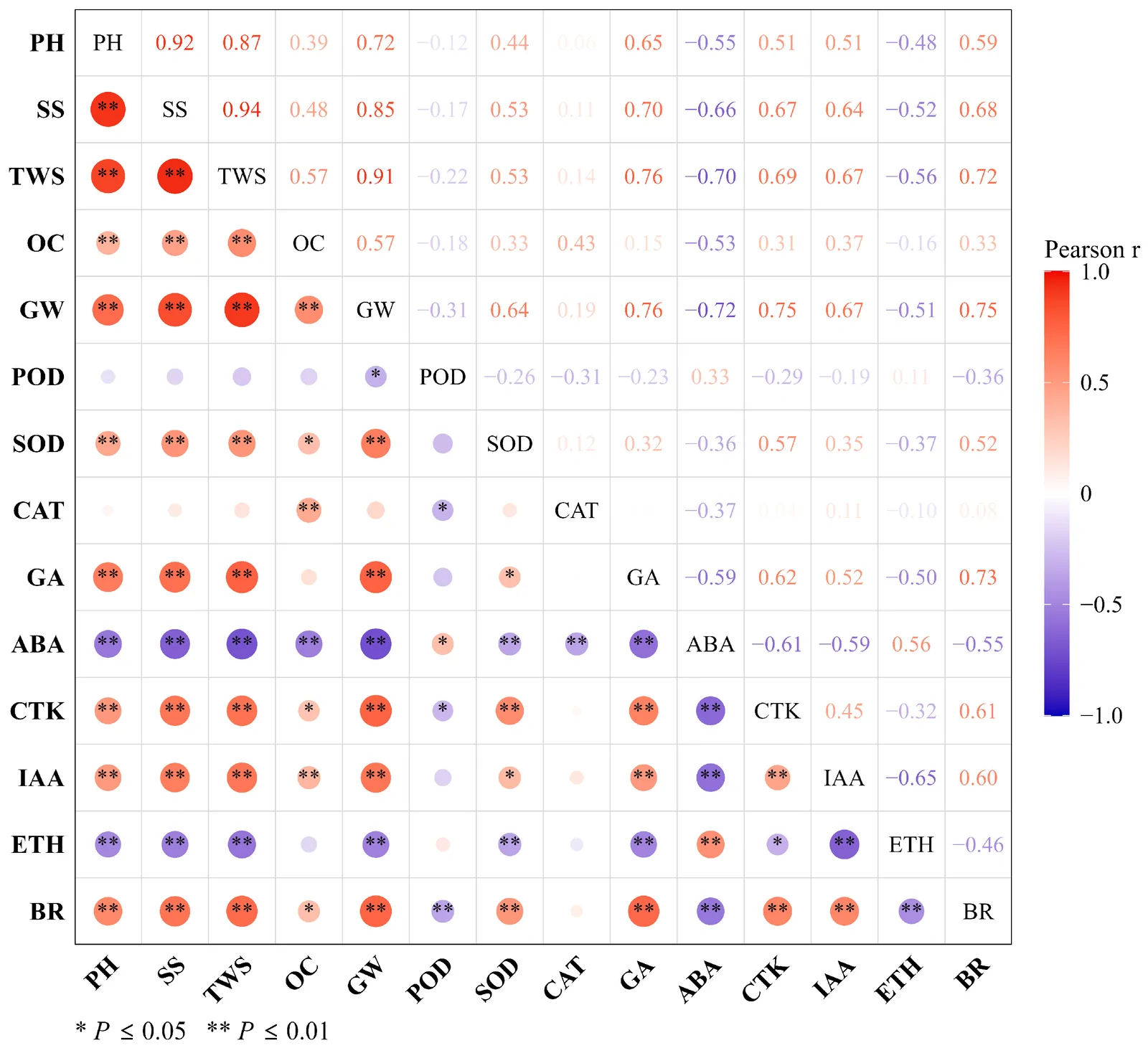
Correlation analysis among main agronomic traits, yield and quality traits, and physiological and biochemical indices in winter turnip rape. PH, plant height; SS, effective silique number per plant; TWS, thousand-seed weight; OC, oil content; GW, seed yield per plant; POD, peroxidase; SOD, superoxide dismutase; CAT, catalase; GA, gibberellin; ABA, abscisic acid; CTK, cytokinin; IAA, auxin; ETH, ethylene; BR, brassinolide. Note: * indicates significance at *p* ≤ 0.05; ** indicates significance at *p* ≤ 0.01.

Pearson correlation analysis was used to reveal the associations among agronomic yield traits, seed quality, antioxidant enzyme activities, and endogenous hormone contents in winter turnip rape with different compatibility (Figure 4). The results showed clear synergistic and antagonistic relationships among rapeseed yield, quality, endogenous hormones, and antioxidant enzymes: growth-promoting hormones (GA, CTK, IAA, and BR) and SOD activity were generally significantly or highly significantly positively correlated with plant height, effective silique number per plant, thousand-seed weight, and seed yield per plant (r = 0.440–0.763). Among these, the correlation coefficients of seed yield per plant with GA, CTK, BR, IAA, and SOD were 0.763, 0.750, 0.748, 0.672, and 0.636, respectively, indicating that elevated GA, CTK, and BR levels were most closely associated with yield improvement. Yield traits were also highly positively correlated with each other: r = 0.941 between silique number and thousand-seed weight, r = 0.923 and 0.873 between plant height and silique number and thousand-seed weight, respectively, r = 0.723, 0.849, and 0.909 between plant height, silique number, thousand-seed weight and seed yield per plant, respectively, and r = 0.572, 0.574, and 0.479 between oil content and thousand-seed weight, seed yield per plant, and silique number, respectively, indicating a synergistic basis for high yield and good quality. Conversely, ABA and ETH were highly significantly negatively correlated with seed yield per plant (r = −0.721 and −0.514) and with plant height, silique number, and thousand-seed weight (r = −0.700 to −0.483). Oil content was correlated with ABA at r = −0.527, indicating that senescence-promoting hormones are detrimental to yield and oil accumulation. CAT was correlated with oil content at r = 0.431. POD was correlated with seed yield per plant at r = −0.314 and with CTK, BR, and CAT at r = −0.291, −0.361, and −0.305, respectively. SOD was correlated with CTK, BR, GA, and IAA at r = 0.318–0.572 and with ABA and ETH at r = −0.362 and −0.369. Among hormones, GA, CTK, IAA, and BR were positively correlated with each other (r = 0.454–0.728). ABA and ETH were positively correlated (r = 0.556), but ABA was correlated with growth-promoting hormones at r = −0.611 to −0.551, and ETH was correlated with IAA, GA, BR, and CTK at r = −0.645, −0.504, −0.459, and −0.319, respectively, reflecting clear antagonism. Oil content was correlated with IAA, BR, SOD, CTK, and CAT at only r = 0.367, 0.330, 0.326, 0.309, and 0.431, respectively, and with ABA at r = −0.527. The correlations were overall weaker than those for yield traits, indicating that quality is less affected by physiological fluctuations. Overall, higher levels of growth-promoting hormones and SOD activity, and lower accumulation of ABA, ETH, and POD, are important physiological characteristics for good growth and high yield in winter turnip rape. CAT, however, was only weakly and non-significantly correlated with yield traits (it was associated only with oil content, r = 0.431), so it should not be grouped with the yield-related indicators.

### 2.4. Structural Equation Model Analysis of the Physiology–Yield Regulatory Network

**Figure 5:**
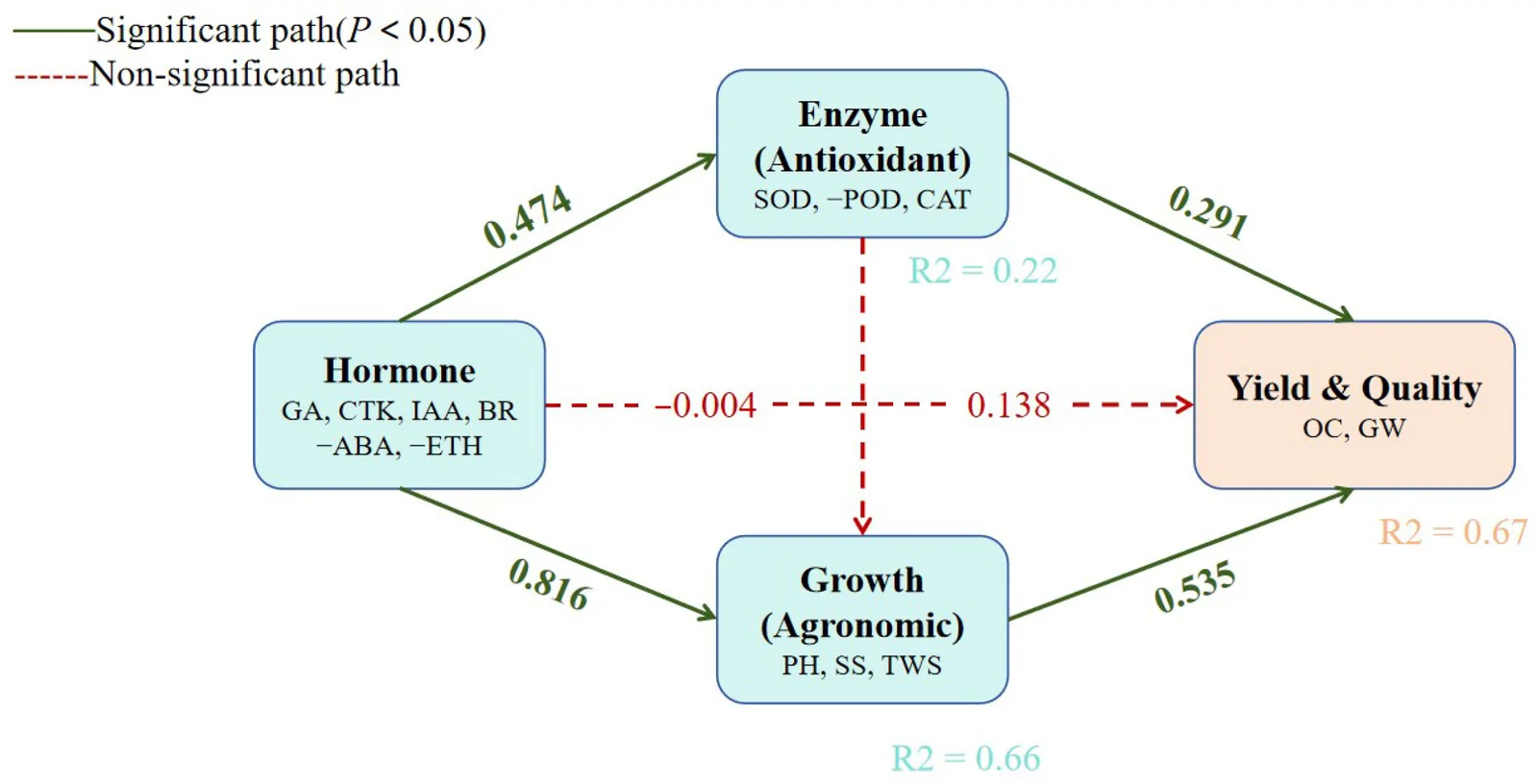
Path diagram of the structural equation model (SEM) for “hormone → enzyme → growth → yield and quality” in winter turnip rape. Values along the arrows are standardized path coefficients. Solid lines indicate significant paths (*p* < 0.05) and dashed lines indicate non-significant paths. R2 in the rectangles denotes the proportion of variance explained for each endogenous variable. H, E, G, and Y denote composite scores of hormone, enzyme activity, growth, and yield and quality, respectively. POD, ABA, and ETH were incorporated into the composite scores in the reverse direction (−POD, −ABA, and −ETH).

Based on the above significant correlation results, a structural equation model (SEM) was further constructed to explore the statistical associations among endogenous hormones, antioxidant metabolism, vegetative growth, and yield and quality (Figure 5). Indicators at each level were first Z-score standardized, then averaged after reversing the sign of the negative-direction indicators (−POD, −ABA, and −ETH), so as to synthesize one composite score per level. Because each level entered the model as a single aggregated score, the contribution of its individual components (e.g., a particular hormone or enzyme) cannot be distinguished. The hormone (H), enzyme activity (E), growth (G), and yield and quality (Y) composite scores were used as manifest variables to construct a four-level recursive path model of “endogenous hormones → antioxidant enzyme activity → growth → yield and quality.” The model explained 22.4%, 66.2%, and 66.8% of the variance in enzyme activity, growth, and yield and quality, respectively (R^2^ = 0.224, 0.662, and 0.668), indicating that this framework can explain a substantial proportion of the variation in yield and quality.

Path analysis results showed that hormones had significant positive direct effects on both enzyme activity (H → E = 0.474, *p* < 0.05) and growth (H → G = 0.816, *p* < 0.05), with the strongest effect on growth. Growth had the largest direct effect on yield and quality (G → Y = 0.535, *p* < 0.05), and enzyme activity also had a significant positive direct effect on yield and quality (E → Y = 0.291, *p* < 0.05). In contrast, the direct effects of enzyme activity on growth (E → G = −0.004, *p* > 0.05) and of hormones on yield and quality (H → Y = 0.138, *p* > 0.05) were both non-significant. These results indicate that the hormone composite is strongly associated with the growth composite, and its association with yield and quality is largely indirect (mediated by the growth composite); however, because the hormone variable is an aggregated score, the model cannot attribute this path to specific hormones, such as GA, CTK, IAA, or BR. Growth thus becomes the core mediating variable linking the hormone composite to yield and quality. The enzyme composite was directly associated with the yield-and-quality composite, but the model does not establish that this reflects maintenance of redox homeostasis or assurance of seed development, nor can it distinguish the contributions of POD, SOD, and CAT. These mechanistic interpretations, therefore, remain hypotheses rather than conclusions demonstrated by the model.

## 3. Discussion

### 3.1. Effects of Self-Compatibility on Growth, Development, Yield, and Quality of Winter Turnip Rape

Self-compatibility is a key trait determining the reproductive mode and fitness of winter turnip rape, and its differences directly affect plant vegetative growth, seed-setting traits, and seed quality formation. This study found that the inhibitory effects of selfing on seedling vegetative growth, agronomic traits, quality, and biomass were far lower in highly self-compatible lines (HSC) than in self-incompatible lines (SI). The mean inbreeding depression rates of major traits in the three types were HSC (4.32%) < SC (8.14%) < SI (20.05%), indicating that the higher the self-compatibility, the stronger the plant’s ability to tolerate selfing and maintain trait stability. This result is consistent with the findings of Song [9] and Qi et al. [26] in turnip rape, namely, that self-compatible materials can alleviate the adverse effects of inbreeding depression on growth and development by ensuring the successful completion of self-pollination and fertilization.

Analysis of seedling leaf traits showed that within the same line, leaf length, leaf width, and leaf number of open-pollinated plants were all higher than those of bagged self-pollinated plants, and the depression of leaf traits after selfing was most significant in SI lines (leaf length IDR reaching 25.81%), indicating that selfing has an obvious inhibitory effect on the early vegetative growth of winter turnip rape. This is consistent with the weakened seedling growth vigor observed by Dong et al. [33] in selfed progenies of winter turnip rape. The IDR of all seedling traits in HSC lines was below 8%, indicating strong selfing adaptability and suggesting that highly compatible materials can still maintain relatively vigorous vegetative growth under selfing conditions, laying a material foundation for later reproductive growth.

Regarding yield component traits, in this study the inbreeding depression rates of effective silique number per plant, seeds per silique, and thousand-seed weight in HSC lines were 3.03%, 11.54%, and 5.05%, respectively, whereas those in SI lines were as high as 20.53%, 23.55%, and 15.64%. Line-level ANOVA and Tukey tests (3 lines per group; 18 line–treatment combinations) showed that the group differences were significant (*p* < 0.05): the SI IDR for effective silique number and thousand-seed weight was significantly higher than that of HSC and SC, which formed one group, so the contrast is a statistically supported difference rather than merely a descriptive trend. Seeds per silique, as a key factor in yield formation, had a CV as high as 82.58% in SI lines, indicating extremely poor trait stability—closely related to the low pollen germination rate, impeded fertilization, and high ovule abortion rate after self-pollination in self-incompatible lines [34]. Wang et al. [35] also found in strong-winter turnip rape that the seeds per silique and yield per plant of self-compatible lines were significantly higher than those of incompatible lines, indicating that self-compatibility can directly contribute to yield formation by improving seed-setting efficiency.

Regarding quality traits, after selfing, oil content decreased by 14.17% and protein by 12.12% in SI lines, with depression magnitudes far greater than in HSC lines (oil content decreased by 3.51% and protein by 4.55%). Qi et al. [26] showed that inter-line hybridization of turnip rape can significantly increase oil content and protein content, which indirectly confirms the negative impact of inbreeding depression on quality. The stable quality traits of self-compatible lines may be due to their ability to maintain normal carbon and nitrogen metabolism and fatty acid synthesis pathways, reducing quality deterioration caused by imbalanced photosynthate allocation during selfing [36]. Biomass analysis further confirmed that the depression rates of grain, stem, pod shell, and root weight after selfing were all significantly higher in SI lines than in HSC lines, with the root-weight IDR reaching 41.77%, indicating that self-incompatibility has a comprehensive inhibitory effect on overall dry matter accumulation, with the root system as the absorptive organ being the first to be affected.

### 3.2. Effects of Self-Compatibility on Physiological and Biochemical Characteristics of Winter Turnip Rape: Antioxidant Enzyme Coordination and Endogenous Hormone Homeostasis

Selfing can lead to reactive oxygen species (ROS) accumulation and oxidative stress, and the antioxidant enzyme system is a key defense line for scavenging ROS and maintaining redox balance. This study found that after selfing, POD activity was elevated and high in SI-SP, SOD was low, but CAT was low only under SI-SP and recovered under SI-OP to the same statistical group as HSC and SC. The three enzymes, therefore, showed altered relative coordination rather than functional disruption of the antioxidant system, and the POD increase is more accurately described as elevated than as “abnormally elevated.” Under open pollination, HSC lines showed significantly increased SOD activity, whereas CAT activity did not increase significantly (OP and SP shared a statistical group), forming a SOD-centered, relatively efficient antioxidant defense that can promptly scavenge ROS generated during selfing or outcrossing and reduce oxidative damage. This is consistent with the view proposed by Dong et al. [33] that self-compatible lines can maintain higher antioxidant capacity and also corroborates reports of decreased antioxidant enzyme activities during inbreeding depression in species such as potato [37], Yali pear [38], and *Eruca sativa* [39].

Endogenous hormone balance is the core signaling network regulating plant growth and development. This study found that the contents of growth-promoting hormones such as GA, CTK, IAA, and BR in HSC lines were significantly higher than in SI lines, whereas the contents of growth-inhibiting and senescence-promoting hormones such as ABA and ETH were generally lower than in SI lines. This difference was significant for ABA and for ETH under SP, but ETH did not differ significantly between HSC-SP and SI-OP, which shared a statistical group. After selfing, ABA and ETH further accumulated in SI lines, while growth-promoting hormones remained at low levels, indicating severe imbalance in their hormone metabolism. Yang et al. [40] also found in lily self-incompatibility research that the incompatibility response was accompanied by a large increase in ABA and a decrease in IAA, consistent with the results of this study. Excessive accumulation of ABA and ETH can inhibit pollen tube elongation and accelerate tissue senescence, which may be an important physiological reason for the low seed-setting rate and weak growth vigor of SI lines after selfing.

Notably, under bagged self-pollination, HSC lines still maintained high levels of growth-promoting hormones, such as GA and CTK, and ABA did not accumulate significantly, indicating that highly self-compatible materials can actively maintain hormone homeostasis in a selfing environment and avoid excessive activation of growth-inhibiting signals. This hormone regulatory ability enables them to maintain the hormone environment required for pollen germination, pollen tube elongation, and seed development after self-pollination, which is also an important physiological basis for their high selfed seed-setting rate and mild depression.

### 3.3. Coupling Relationships Among Agronomic–Quality–Physiological–Hormone Traits and the Yield-Quality Regulatory Network

Correlation analysis extends single-factor comparisons to multi-trait coupling networks, helping to identify key physiological indicators through which self-compatibility affects yield and quality from a holistic perspective. In this study, growth-promoting hormones such as GA, CTK, IAA, and BR were significantly or highly significantly positively correlated with seed yield per plant, effective silique number per plant, thousand-seed weight, and plant height, with GA, CTK, and BR having the highest correlation coefficients with seed yield. This is consistent with the conclusion of de Bouillé et al. [41] in studies of silique development in oilseed rape (*Brassica napus*), namely, that cytokinin and IAA levels in early silique establishment directly determine silique number, seeds per silique, and seed weight, whereas elevated ABA and decreased IAA and cytokinin accompany silique maturation. Among yield component traits, effective silique number per plant, thousand-seed weight, and seed yield per plant were highly positively correlated, consistent with the correlation analysis results of Li et al. [42] in winter rapeseed in the arid and cold regions of northern China, Tsering et al. [43] in spring turnip rape in Tibet, and Yang et al. [44] in oilseed rape, indicating that source–sink traits, such as plant type, silique setting, and grain filling, are synergistically improved in high-yield materials. Oil content was positively correlated with thousand-seed weight, seed yield per plant, IAA, CTK, SOD, and CAT, and negatively correlated with ABA, consistent with the finding of Guo et al. [45] that higher ZR and IAA in the early and middle stages of seed development are conducive to oil and oleic acid accumulation. However, the correlations between oil content and various physiological and hormone indicators were overall weaker than those for yield traits, indicating that oil accumulation is more controlled by genetic background and relatively less affected by physiological fluctuations caused by pollination method and compatibility differences, which also explains the phenomenon in this study that the inbreeding depression rates of quality traits were overall lower than those of yield traits.

Meanwhile, ABA and ETH were highly significantly negatively correlated with seed yield per plant and major silique traits, and ABA and ETH were highly significantly positively correlated with each other and generally significantly or highly significantly negatively correlated with the four growth-promoting hormones, suggesting an obvious antagonistic balance between growth-promoting and senescence-promoting hormones, consistent with the view of Bartoli et al. [46] that hormone signaling and redox signaling are tightly coupled in controlling growth and cross-tolerance to stress. Notably, POD activity was negatively (but not significantly) correlated with seed yield per plant (r = −0.314) and negatively correlated with CTK and BR contents and CAT activity, whereas SOD was positively correlated with yield and growth-promoting hormones and CAT showed only weak, non-significant correlations with yield. This suggests that the high POD activity in SI lines may be associated with tissue stress and senescence rather than with effective antioxidant capacity, but this is an interpretation rather than direct evidence. Without direct ROS or oxidative-damage measurements, and given that CAT was not consistently low, the idea that a single-POD pathway compensates for deficient SOD–CAT scavenging should be regarded as a hypothesis, and the antioxidant system should be described as altered rather than disrupted.

To further distinguish the direct and indirect effects of each physiological factor on yield and quality, this study constructed a recursive structural equation model of “hormone → enzyme activity → growth → yield and quality.” Structural equation modeling can decompose simple correlations into direct and indirect effects and is an effective tool for dissecting multivariate cascade relationships in crop yield formation [32]. The results showed that the direct path coefficient of hormones on growth was the largest (0.816), the direct effect of growth on yield and quality was the largest (0.535), and the direct effect of hormones on yield and quality was non-significant (0.138), indicating that endogenous hormones are the most important upstream drivers of growth and development, and their effects on yield and quality are mainly realized indirectly by promoting vegetative growth and reproductive organ establishment. Vegetative growth thus becomes the core mediating variable linking hormones to yield and quality. This is consistent with the findings of Wang et al. [47], who through path analysis found that under drought, exogenous hormones mainly indirectly affect sweet potato yield through growth processes, such as net photosynthetic rate, and of Miao et al. [31], who used structural equation modeling to reveal that water requirement and photosynthetic amount in rice mainly determine yield through the indirect effects of population quality and yield component factors—together supporting the yield formation pattern of “source (vegetative growth)–sink (grain) interaction, with upstream factors acting mainly through indirect effects.”

Unlike the hormone pathway, the direct path of antioxidant enzymes on vegetative growth was non-significant (−0.004), but they had a significant positive direct effect on yield and quality (0.291), indicating that the enzyme composite is associated with yield and quality rather than acting through vegetative growth. This is broadly consistent with the results of Zhou et al. [48], who through structural equation modeling in maize on saline–alkali land found that under moderate salt stress yield was directly related to growth, photosynthesis, and CAT activity, of Xu et al. [49], who used partial least squares structural equation modeling to identify an “endogenous hormones–antioxidant enzymes–photosynthesis–yield” pathway, and of He et al. [50], who reported that calcium and magnesium relate to kernel sugar accumulation through a hormone–CAT pathway. However, these literature parallels do not turn the present paths into demonstrated mechanisms: because the enzyme variable in this study is a composite score, the model cannot confirm SOD–CAT synergistic action, redox homeostasis maintenance, or oxidative damage as the causal process, and the SI pattern is more cautiously stated as altered enzyme coordination together with severe depression than as demonstrated oxidative damage to reproductive organs. Combining the two pathways, highly self-compatible materials tend to maintain higher levels of GA, CTK, IAA, and BR alongside relatively stable enzyme coordination, supporting stable yield and good quality under selfing, whereas SI lines show weaker growth-promoting hormone signals, more ABA and ETH, and altered enzyme coordination together with severe depression. It should be noted that this study has a limited sample size and is based on a single year and location; synthesizing multiple indicators into composite scores, while highlighting the dominant pathways, also loses indicator-specific information, and correlations do not equate to causation. The molecular mechanism by which self-compatibility couples S-locus signaling with the hormone–redox network to regulate yield still needs to be verified through multi-year and multi-location experiments combined with targeted regulation of hormones and enzymes and molecular evidence.

### 3.4. Limitations of the Study

Several limitations of the present study should be acknowledged. First, the sample size was limited (18 line–treatment combinations; 9 lines) and the field experiment was conducted at a single location in a single year, so the generality of the findings across environments and years remains to be confirmed. Second, only three lines (genotypes) represented each group (the IDR tests used an error term with 6 df), which limited statistical power; consequently, the SC–SI contrast did not reach significance for oil content, seed yield, and biomass, and the antioxidant enzyme and hormone data were collected at a single developmental stage. Third, the composite scores used in the SEM, while highlighting dominant pathways, lose indicator-specific information, and the correlation and path analyses establish association rather than causation. Finally, this study did not directly measure ROS levels, oxidative damage, or stress tolerance; therefore, the mechanistic links among S-locus signaling, the hormone–redox network, and yield formation remain hypotheses that need to be tested through dedicated experiments.

## 4. Materials and Methods

### 4.1. Experimental Materials

The experimental materials were nine winter turnip rape lines (three per compatibility type) provided by the Rapeseed Research Group of Gansu Agricultural University (Table 6). Each line was evaluated under self-pollination and open pollination, giving 18 line–treatment combinations. Their self-compatibility was previously determined through bagged self-pollination experiments based on the relative self-compatibility index (RSCI) [28].

### 4.2. Field Design and Pollination Treatments

The experimental materials were planted in August 2023 at the Rapeseed Experimental Base in Lanzhou New Area, Gansu Province (103°70′ E, 37°00′ N). Single-row planting was adopted, with a row length of 2 m, plant spacing of 20 cm, and 20 plants per material. At the full-bloom stage (defined as the stage when approximately 50% of the flowers on the main raceme and primary branches had opened, while unopened buds remained clustered at the raceme apex), robust individual plants were selected, and 20 flower buds on branches were bagged for self-pollination (SP) and labeled with tags. The selected buds were at the balloon stage—plump, unopened buds approximately 1–2 days before anthesis, with the petals still fully enclosed by the sepals—which is the standard stage for controlled self-pollination, and the remaining flowers were subjected to open pollination (OP). At maturity (defined as the stage when approximately 70–80% of the siliques on the plant had turned yellow–brown, the seeds had attained their mature black/brown color and rattled within the pod when shaken, and the lower and middle siliques were fully dry while the stem remained partly green), the labeled bagged self-pollinated siliques and open-pollinated siliques were harvested separately, and the number of set siliques, seeds per silique, and silique length were counted. In 2024, the selfed and open-pollinated progenies of nine lines were replanted under the same management practices. At the seedling stage, five plants per treatment were randomly selected and labeled for the determination of botanical traits; at maturity, agronomic traits were determined, and seed quality was measured after harvest.

### 4.3. Self-Compatibility Classification and Calculation of Inbreeding Depression Rate

The self-compatibility index (SCI, i.e., the number of seeds set per self-pollinated flower) was used as the basic criterion, and the relative self-compatibility index (RSCI) was introduced to classify the materials into highly self-compatible type (0.50 < RSCI ≤ 1.00), self-compatible type (0.20 < RSCI ≤ 0.50), and self-incompatible type (0.10 < RSCI ≤ 0.20). The inbreeding depression rate was calculated as follows:

IDR = (Wo − Ws)/Wo × 100%,

where Ws is the mean trait value of the selfed (SP) progeny population and Wo is the mean trait value of the open-pollinated (OP) progeny population.

Line-level IDR values for the six core traits were compared among the HSC, SC, and SI groups by one-way analysis of variance (ANOVA) followed by Tukey’s honestly significant difference (HSD) multiple-comparison test at *p* < 0.05. The line, rather than the individual plant, was treated as the independent replicate (*n* = 3 lines per group; 18 line–treatment combinations in total), so that this test is equivalent to testing the group × pollination treatment interaction. The analyses were performed in Python 3.9 using the SciPy 1.10.1 and statsmodels packages 0.13.5.

### 4.4. Trait Determination

Referring to the “Recording Specification for Variety Trials of Northern Winter Turnip Rape” (DB62/T 2643—2016) and the method of Wang [35], traits such as leaf length (cm), leaf width (cm), petiole length (cm), leaf number (No.), number of lobed leaf pairs (pairs), and rootstock diameter (mm) were recorded at the seedling stage. At maturity, 5 labeled plants per treatment were sampled to assess root diameter, root length, shortened stem end, plant height, branching point height, number of primary branches, effective length and effective silique number of the main inflorescence, effective silique number per plant, silique length, seeds per silique, and thousand-seed weight, and the dry weights of grain, stem, pod shell, and root were determined. After natural air-drying, threshing, and cleaning, the seeds were analyzed for protein, oil content, stearic acid, oleic acid, linoleic acid, linolenic acid, erucic acid, and glucosinolate contents using a FOSS NIRS DS3 F near-infrared spectrometer (FOSS, Suzhou, China).

### 4.5. Determination of Antioxidant Enzyme Activities

Fresh leaves were collected, the main veins were removed, and the samples were immediately frozen in liquid nitrogen and stored at −80 °C. Superoxide dismutase (SOD, EC 1.15.1.1), catalase (CAT, EC 1.11.1.6), and peroxidase (POD, EC 1.11.1.7) activities were determined using assay kits (BC0175 for SOD, BC0205 for CAT, and a peroxidase kit for POD; Solarbio, Beijing Solarbio Science & Technology Co., Ltd., Beijing, China), with all operations performed according to the manufacturer’s instructions, following the method of Dong et al. [33]. SOD activity is expressed as U/g FW, and POD and CAT activities are expressed as U/(g·min).

### 4.6. Determination of Endogenous Hormone Contents

Samples were collected at the flowering stage, frozen in liquid nitrogen, and stored at −80 °C. The contents of auxin (IAA), gibberellin (GA), cytokinin (CTK), abscisic acid (ABA), ethylene (ETH), and brassinolide (BR) were determined by enzyme-linked immunosorbent assay (ELISA) [51], with 3 replicates per treatment. Briefly, 1 g of fresh sample was ground in an ice bath with 1 mL of 80% methanol (containing 1 mmol/L BHT) and extracted at 4 °C for 1 h. After centrifugation at 4000 r/min for 5 min, the supernatant was collected, evaporated to dryness under reduced pressure, redissolved in pH 2.8 purified water with a small amount of PVP, extracted three times with diethyl ether, and then evaporated to dryness and brought to volume. The microplate was coated with hormone antigen at 4 °C overnight. After washing, standards or samples and primary antibody were added and incubated at room temperature for 2 h, followed by the addition of enzyme-labeled secondary antibody and incubation for 1 h. After substrate color development, the reaction was terminated with 6 mol/L sulfuric acid (H_2_SO_4_), and the optical density (OD) was measured at a wavelength of 400 nm. Hormone contents were calculated by logarithmic transformation and linear regression against the standard curve.

### 4.7. Data Processing

Data were organized in Excel. One-way analysis of variance (ANOVA) followed by Duncan’s new multiple-range test (*p* < 0.05) was performed in SPSS (31.0.2) for the treatment comparisons in Table 1, Table 2, Table 3 and Table 4 and Figure 2 and Figure 3. Line-level IDR values were compared among compatibility groups by one-way ANOVA followed by Tukey’s HSD test in Python (SciPy and statsmodels), as described in Section 4.3. A recursive structural equation model was constructed based on the Pearson correlation matrix of 14 indicators. Each level was first Z-score standardized and synthesized into a composite score in the specified direction, and then the path model was fitted by maximum likelihood estimation to estimate standardized path coefficients, direct effects, indirect effects, and total effects. The 95% confidence intervals of effects were estimated by 2000 bootstrap resamples, and model fit was evaluated by χ^2^, RMSEA, CFI, and SRMR, with *p* < 0.05 as the criterion for path significance. Statistical analyses were performed in Python 3 and R 4.x (lavaan and semPlot packages).

## 5. Conclusions

The degree of inbreeding depression in winter turnip rape was negatively associated with self-compatibility. The mean inbreeding depression rates of major traits in HSC, SC, and SI lines were 4.32%, 8.14%, and 20.05%. Line-level ANOVA and Tukey tests confirmed that the IDRs of SI lines were significantly higher than those of HSC lines for all six core traits, with SC lines intermediate; the largest numerical differences were found in seed yield, effective silique number per plant, and biomass. These results indicate that the higher the self-compatibility, the stronger the plant’s ability to tolerate selfing and maintain stable growth and yield. Self-compatible lines showed relatively stable hormone balance and enzyme coordination: HSC lines maintained higher SOD activity and higher contents of growth-promoting hormones, such as GA, CTK, and IAA, whereas CAT did not show a consistent difference; after selfing, SI lines showed altered enzyme coordination and accumulation of ABA, while the ETH differences were not consistently significant. Correlation analysis showed that GA, CTK, IAA, and BR were significantly positively correlated with seed yield per plant, effective silique number per plant, and thousand-seed weight, whereas their correlations with oil content were not significant. ABA, ETH, and POD activity were negatively (and for ABA/ETH significantly) correlated with yield. The structural equation model further showed that the hormone composite was associated with yield and quality mainly indirectly through the growth composite (path coefficients of hormone → growth and growth → yield and quality were 0.816 and 0.535, respectively), whereas the enzyme composite was directly associated with yield and quality (0.291). These are associations among composite scores and do not confirm redox homeostasis or grain-filling mechanisms. The highly self-compatible materials alleviate inbreeding depression and achieve stable yield and good quality through the indirect, growth-mediated pathway and the direct enzyme-associated pathway, providing a theoretical basis for the screening of self-compatible germplasm and for high-yield breeding of winter turnip rape. Stress-resistant breeding was not examined in this study and would require direct stress-tolerance assays. Future work should expand the panel and conduct multi-year, multi-location field trials to verify the stability of the observed effects, and combine transcriptomic, proteomic, and metabolomic analyses with targeted hormone and enzyme manipulation to elucidate the molecular pathways linking S-locus signaling to the hormone–redox network and yield formation. These efforts will further support the breeding and application of highly self-compatible winter turnip rape varieties.

## Figures and Tables

**Table 1:** Vegetative growth traits at the seedling stage for HSC, SC, and SI lines under self-pollination (SP) and open pollination (OP).

Line	Index	Leaf Length (cm)	Leaf Width (cm)	Number of Leaves
HSC-SP	Mean ± SD	4.71 ± 1.06 b	4.03 ± 0.74 b	5.52 ± 0.91 ab
	CV (%)	22.51	18.36	16.49
HSC-OP	Mean ± SD	4.89 ± 1.37 a	4.38 ± 0.99 a	5.72 ± 1.65 a
	CV (%)	28.02	22.60	28.85
HSC	IDR (%)	3.68	7.99	3.50
SC-SP	Mean ± SD	3.21 ± 0.83 c	3.32 ± 0.84 c	5.00 ± 1.73 bc
	CV (%)	25.86	25.30	34.60
SC-OP	Mean ± SD	3.40 ± 1.01 c	3.66 ± 0.41 b	5.32 ± 1.61 bc
	CV (%)	29.71	11.20	31.45
SC	IDR (%)	5.59	9.29	6.01
SI-SP	Mean ± SD	2.76 ± 0.72 d	2.54 ± 0.57 d	4.56 ± 0.77 c
	CV (%)	26.09	22.44	16.89
SI-OP	Mean ± SD	3.72 ± 1.36 c	3.27 ± 0.68 c	5.26 ± 2.11 b
	CV (%)	36.56	20.80	40.11
SI	IDR (%)	25.81	22.32	13.31

Note: HSC, highly self-compatible; SC, self-compatible; SI, self-incompatible; SP, self-pollination (bagged); OP, open pollination; CV, coefficient of variation; IDR, inbreeding depression rate. Different letters within a column indicate significant differences (*p* < 0.05).

**Table 2:** Main agronomic traits of HSC, SC, and SI lines under SP and OP.

Line	Index	Plant Height (cm)	Branching Point (cm)	Primary Branches (No.)	Main Inflor. Length (cm)	Effective Siliques (No./Plant)	Silique Length (cm)	Seeds per Silique	Thousand-Seed Weight (g)
HSC-SP	Mean ± SD	106.78 ± 7.08 ab	15.95 ± 12.46 b	8.56 ± 2.03 ab	39.12 ± 4.02 bc	196.37 ± 8.40 a	7.27 ± 0.55 b	15.33 ± 6.39 b	3.01 ± 0.14 b
	CV (%)	6.63	78.12	23.72	10.28	4.28	7.57	41.68	4.66
HSC-OP	Mean ± SD	110.50 ± 8.50 a	18.00 ± 5.50 a	9.80 ± 1.00 a	44.20 ± 7.50 a	202.50 ± 10.20 a	8.46 ± 0.78 a	17.33 ± 5.21 a	3.17 ± 0.02 a
	CV (%)	7.69	30.56	10.2	16.97	5.04	9.22	30.06	0.73
HSC	IDR (%)	3.37	11.39	12.56	11.49	3.03	14.07	11.54	5.05
SC-SP	Mean ± SD	92.90 ± 6.50 cd	8.30 ± 2.90 cd	6.80 ± 1.60 cd	34.50 ± 9.50 cd	154.40 ± 22.50 bc	4.19 ± 0.25 de	14.50 ± 4.03 b	2.44 ± 0.04 d
	CV (%)	7.00	27.54	23.53	27.54	14.57	5.97	27.79	1.64
SC-OP	Mean ± SD	97.10 ± 7.20 bc	9.90 ± 3.80 c	8.30 ± 1.90 c	42.60 ± 5.20 ab	162.90 ± 25.30 b	5.45 ± 0.28 c	17.28 ± 4.77 a	2.63 ± 0.15 c
	CV (%)	7.42	22.89	22.89	12.21	15.53	5.14	27.60	5.77
SC	IDR (%)	4.33	18.07	18.07	19.01	5.22	23.12	16.09	7.22
SI-SP	Mean ± SD	86.50 ± 5.80 e	4.60 ± 3.10 e	4.30 ± 1.20 d	27.10 ± 5.00 e	114.60 ± 12.80 d	3.66 ± 0.34 d	5.11 ± 3.22 d	2.05 ± 0.08 e
	CV (%)	6.71	27.91	27.91	18.45	11.17	9.29	82.58	4.06
SI-OP	Mean ± SD	93.20 ± 6.30 cd	5.80 ± 1.70 e	7.00 ± 1.30 c	39.80 ± 12.50 bc	144.20 ± 13.50 c	4.54 ± 1.12 e	11.00 ± 3.86 c	2.43 ± 0.05 d
	CV (%)	6.76	18.57	18.57	31.41	9.36	24.67	35.09	1.98
SI	IDR (%)	7.19	38.57	38.57	31.91	20.53	19.38	23.55	15.64

Note: HSC, highly self-compatible; SC, self-compatible; SI, self-incompatible; SP, self-pollination (bagged); OP, open pollination; CV, coefficient of variation; IDR, inbreeding depression rate; SD, standard deviation. Different letters within a column indicate significant differences (*p* < 0.05).

**Table 3:** Main seed quality traits of HSC, SC, and SI lines under SP and OP.

Line	Index	Protein (%)	Oil Content (%)	Oleic Acid (%)	Linolenic Acid (%)	Erucic Acid (%)	Glucosinolates (µmol/g)
HSC-SP	Mean ± SD	26.25 ± 1.08 a	42.92 ± 0.43 b	30.02 ± 3.20 b	8.63 ± 0.33 b	38.51 ± 0.77 ab	99.13 ± 4.09 b
	CV (%)	4.11	1.00	10.66	3.82	2.00	4.13
HSC-OP	Mean ± SD	27.50 ± 0.35 a	44.48 ± 0.58 a	31.53 ± 3.41 a	9.15 ± 0.22 a	39.02 ± 1.83 a	111.93 ± 6.22 a
	CV (%)	1.27	1.30	10.82	2.40	4.69	5.56
HSC	IDR (%)	4.55	3.51	4.79	5.68	1.31	11.44
SC-SP	Mean ± SD	24.38 ± 0.92 b	41.52 ± 0.95 c	29.33 ± 2.08 b	8.49 ± 0.21 b	35.17 ± 2.35 c	98.18 ± 4.89 b
	CV (%)	3.77	2.29	7.09	2.47	6.68	4.98
SC-OP	Mean ± SD	26.92 ± 0.37 a	45.32 ± 0.96 a	30.63 ± 2.60 b	9.04 ± 0.41 a	36.76 ± 1.80 b	115.08 ± 7.27 a
	CV (%)	1.38	2.12	8.49	4.54	4.90	6.32
SC	IDR (%)	9.44	8.38	4.24	6.08	4.33	14.69
SI-SP	Mean ± SD	22.25 ± 0.53 c	38.15 ± 1.15 d	24.20 ± 0.54 c	7.28 ± 0.23 c	36.68 ± 1.01 b	90.00 ± 3.30 c
	CV (%)	2.38	3.01	2.23	3.16	2.75	3.67
SI-OP	Mean ± SD	25.32 ± 0.44 b	44.45 ± 2.03 a	28.63 ± 2.61 b	8.72 ± 0.16 b	38.79 ± 0.88 a	107.82 ± 2.93 a
	CV (%)	1.74	4.57	9.12	1.84	2.27	2.71
SI	IDR (%)	12.12	14.17	15.47	16.51	5.44	16.53

Note: HSC, highly self-compatible; SC, self-compatible; SI, self-incompatible; SP, self-pollination (bagged); OP, open pollination; CV, coefficient of variation; IDR, inbreeding depression rate; SD, standard deviation. Different letters within a column indicate significant differences (*p* < 0.05).

**Table 4:** Biomass components of HSC, SC, and SI lines under SP and OP.

Line	Index	Grain (g)	Stem (g)	Pod Shell (g)	Root Weight (g)
HSC-SP	Mean ± SD	16.64 ± 0.99 b	18.82 ± 2.10 c	10.65 ± 0.87 b	3.45 ± 0.45 b
	CV (%)	6.33	11.16	8.17	13.04
HSC-OP	Mean ± SD	17.26 ± 1.40 a	21.08 ± 1.62 a	10.86 ± 1.40 a	3.56 ± 0.80 a
	CV (%)	8.11	7.68	12.89	22.47
HSC	IDR (%)	4.69	10.62	1.97	3.1
SC-SP	Mean ± SD	13.08 ± 1.21 c	16.74 ± 4.10 d	8.51 ± 1.14 d	2.72 ± 1.01 d
	CV (%)	9.25	24.49	13.4	37.13
SC-OP	Mean ± SD	14.44 ± 0.03 bc	20.70 ± 0.86 a	9.39 ± 0.96 c	3.19 ± 1.08 c
	CV (%)	0.21	3.79	10.22	33.86
SC	IDR (%)	9.39	19.12	9.47	14.93
SI-SP	Mean ± SD	7.60 ± 0.20 e	11.94 ± 1.11 e	5.61 ± 0.38 e	1.84 ± 0.15 e
	CV (%)	2.36	11.17	6.77	12.1
SI-OP	Mean ± SD	10.52 ± 0.28 d	17.94 ± 4.21 d	7.55 ± 0.96 d	3.16 ± 0.22 c
	CV (%)	2.66	23.47	12.72	7.01
SI	IDR (%)	27.9	35.11	34.58	41.77

Note: HSC, highly self-compatible; SC, self-compatible; SI, self-incompatible; SP, self-pollination (bagged); OP, open pollination; CV, coefficient of variation; IDR, inbreeding depression rate; SD, standard deviation. Different letters within a column indicate significant differences (*p* < 0.05).

**Table 5:** Inbreeding depression rate (IDR, %) of major traits in HSC, SC, and SI lines.

Trait	HSC	SC	SI
Plant height	3.37 ^a^	4.33 ^a^	7.19 ^b^
Effective silique number per plant	3.03 ^a^	5.22 ^a^	20.53 ^b^
Thousand-seed weight	5.05 ^a^	7.22 ^a^	15.64 ^b^
Oil content	3.51 ^a^	8.38 ^ab^	14.17 ^b^
Grain weight	4.69 ^a^	9.39 ^ab^	27.90 ^b^
Biological yield	6.29 ^a^	14.30 ^ab^	34.85 ^b^
Mean	4.32	8.14	20.05

Note: Different letters within a row indicate significant differences among groups at *p* < 0.05 (one-way ANOVA followed by Tukey’s HSD; 3 lines per group; 18 line–treatment combinations).

**Table 6:** Names, sources, and self-compatibility of the 9 experimental lines under the 2 pollination treatments (18 line–treatment combinations).

Line	Source	Compatibility Group	Relative Self-Compatibility Index
S68-2SP	Gansu Agricultural University	Highly self-compatible (HSC)	0.86
S68-2OP			
S116-1SP	Gansu Agricultural University	Highly self-compatible (HSC)	0.78
S116-1OP			
S177-1SP	Gansu Agricultural University	Highly self-compatible (HSC)	0.73
S177-1OP			
S118-1SP	Gansu Agricultural University	Self-compatible (SC)	0.46
S118-1OP			
S135-2SP	Gansu Agricultural University	Self-compatible (SC)	0.48
S135-2OP			
S278-1SP	Gansu Agricultural University	Self-compatible (SC)	0.44
S278-1OP			
S111-1SP	Gansu Agricultural University	Self-incompatible (SI)	0.17
S111-1OP			
S148-1SP	Gansu Agricultural University	Self-incompatible (SI)	0.14
S148-1OP			
S510-2SP	Gansu Agricultural University	Self-incompatible (SI)	0.13
S510-2OP			

## Data Availability

The original contributions presented in this study are included in the article. Further inquiries can be directed to the corresponding authors.
